# Supplementary material for: Enhancing myocardial repair with CardioClusters
Source: Nat Commun. 2020 Aug 7;11:3955. doi: 10.1038/s41467-020-17742-z (PMC7414230; doi:10.1038/s41467-020-17742-z)
Supplement: Supplementary file 1 — Supplementary Information [file 41467_2020_17742_MOESM1_ESM.pdf]

Enhancing Myocardial Repair with CardioClusters

Monsanto et al.

Supplementary Table 1. List of Media

	Component	Catalog Number
Cardiac Interstitial Cell Medium	F12 HAM's (1x)	SH30026.01, HyClone
	10% ES FBS	16141079, Gibco
	1% Penicillin-Streptomycin-Glutamine (100X)	10378016, Gibco
	5 mU/mL human erythropoietin	E5627, Sigma-Aldrich
	10 ng/mL human recombinant basic FGF	HRP-0011, Biopioneer
	0.2 mM L-Glutathione	66013-256, Sigma-Aldrich
Endothelial Progenitor Cell Medium	EBM-2 Basal Medium	CC-3156, Lonza
	EGM-2 Kit Supplements and Growth Factors:<br>• 0.5 mL Human Epidermal Growth Factor<br>• 0.5 mL Insulin-Like Growth Factor-1<br>• 0.5 mL Vascular Endothelial Growth Factor<br>• 0.5 mL HEPARIN<br>• 0.5 mL Gentamicin Sulfate Amphotericin-B<br>• 0.5 mL Ascorbic Acid<br>• 2.0 mL Human Fibroblast Growth Factor-B<br>• 2.0 Hydrocortisone<br>• 10 mL FBS	CC-4176, Lonza
Mesenchymal Stem Cell Medium	10.1 g/L Minimum Essential Medium Eagle, Alpha Modification	M0644, Sigma-Aldrich
	20% FBS	FB-01, Omega Scientific, inc.
	1% Penicillin-Streptomycin-Glutamine (100X)	10378-016, Gibco
	Cell Culture Grade Water	
Basic Buffer	11 g/L Minimum Essential Medium Eagle, Joklik Modification	M0518, Sigma-Aldrich
	3 mM HEPES	H3375, Sigma-Aldrich
	1% Penicillin-Streptomycin-Glutamine (100X)	10378-016, Gibco
	10 mM Taurine	T0625, Sigma-Aldrich
	Insulin, solvate in 3% Acetic Acid/PBS	I-5500, Sigma-Aldrich
	1% Amphotericin B	15290-018, Invitrogen
	50 mg Gentamicin	G1397, Sigma-Aldrich
	Cell Culture Grade Water	

Supplementary Table 2. List of Antibodies

Antibody	Vendor	Catalog Number	Dilution Flow	Dilution ICC/IHC
**C-Kit** (CD117)	R&D systems	AF1356	1:33	-
**Thy-1** (CD90)	Biolegend	328109	1:33	-
**Endoglin** (CD105)	Biolegend	323203	1:33	-
**Prominin-1 (**CD133)	Thermo Fisher Scientific	PA5-38014	1:33	-
**PTPRC (**CD45)	Biolegend	368507	1:33	-
**cTNT,** ALEXA FLUOR® 488 Conjugated	Biocompare	bs-10648R-A488	-	1:200
**Tropomyosin**	Sigma-Aldrich	T 9283	-	1:200
**eGFP**	Molecular Probes	A-11122	-	1:100
**mCherry**	Thermo Fisher Scientific	M11240	-	1:100
**Isolectin GS-IB4,** ALEXA FLUOR® 568 Conjugated	Thermo Fisher Scientific	I21412	-	1:100
**WGA**	Thermo Fisher Scientific	W32465	-	1:500
**Myc tag**	Thermo Fisher Scientific	PA3-981		1:100
**HA-prope**	Santa Cruz Biotechnology	SC-7392	-	1:100
**DAPI (**4′,6-diamidino-2-phenylindole**)**	Sigma-Aldrich	D9542	-	1:10,000
**Phalloidin**	Thermo Fisher Scientific	A12379	-	1:1,000

Supplementary Table 3. List of Primers

Target	Fwd Primer Sequence	Rev Primer Sequence
CD31	CCAAGCCCGAACTGGAATCT	CACTGTCCGACTTTGAGGCT
Desmin (rat)	AGCCTGGGTCAGAGACAGAA	TATCTCCTGCTCCCACATCC
GATA4	CTCAGAAGGCAGAGAGTGTGTCAA	CACAGATAGTGACCCGTCCCAT
HGF	GGCTGGGGCTACACTGGATTG	CCACCATAATCCCCCTCACAT
IGF	GACCGCGGCTTCTACTTCAG	AAGAACTTGCCCACGGGGTAT
IL-6	TCGAGCCCACCGGGAACGAA	GCAGGGAAGGCAGCAGGCAA
SDF-1 (human)	CAGTCAACCTGGGCAAAGCC	AGCTTTGGTCCTGAGAGTCC
SDF-1 (rat)	GTCCTCTTGCTGTCCAGCTC	AGATGCTTGACGTTGGCTCT
SMA	CCCAGCCAAGCACTGTCAGGAATCCT	TCACACACCAAGGCAGTGCTGTCC
18S	CGAGCCGCCTGGATACC	CATGGCCTCAGTTCCGAAAA

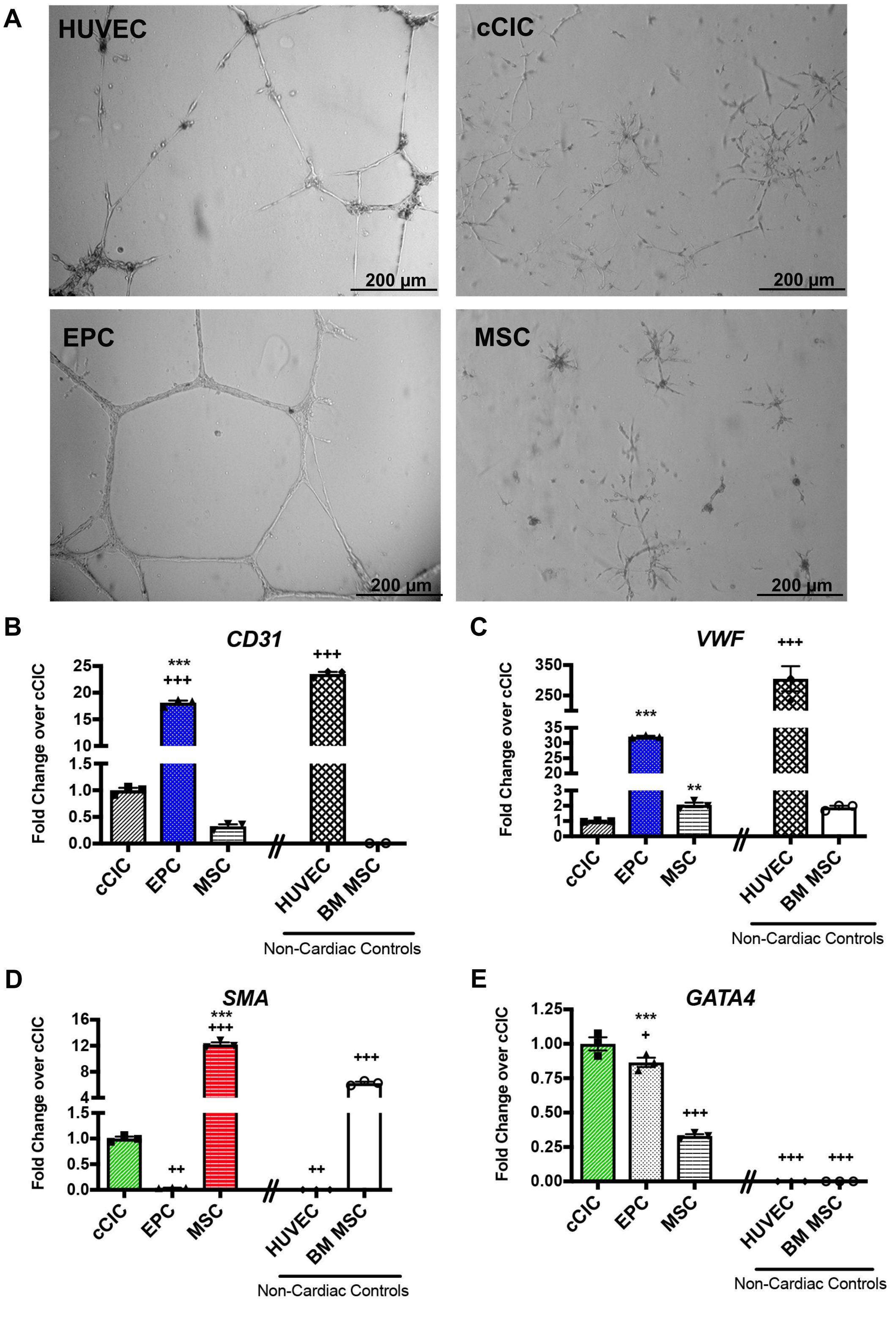

Supplementary Figure 1. *In vitro* lineage assessment relative to non-cardiac controls HUVECs and BM MSCs
A, Representative images of tubular network formation when plated on growth factor reduced matrigel for HUVEC, cCIC, EPC, and MSC. Scale bar, 200 μm. **B–E**, Bar graphs using established cell lines, HUVEC and bone marrow-derived MSC (BM MSC), to assess the potential of cardiac stem cells to commit to an angiogenic **(B, C)**, smooth muscle **(D)**, and cardiogenic **(E)** fate. Data represent mean (n=FH-09 heart [cell line used to create CardioClusters], run in triplicate) ± SEM. Data are presented as 1-way ANOVA with Dunnett's comparison test, (**B**) $^{+++}$p<0.001, ***p<0.001, (**C**) $^{+++}$p<0.001, **p=0.007, ***p<0.001, (**D**) $^{++}$p=0.009 [EPC], $^{++}$p=0.007 [HUVEC], $^{+++}$p<0.001, *p=0.018, ***p<0.001, (**E**) $^{+}$p=0.017, ***p<0.001, $^{+++}$p<0.001, versus cCIC. *excludes HUVEC and BM MSC from statistical analysis, $^{+}$includes HUVEC and BM MSC in statistical analysis. GATA4 indicates GATA binding protein 4; PECAM-1, platelet endothelial cell adhesion molecule; SMA, α-smooth muscle actin; and VWF, von Willebrand factor.

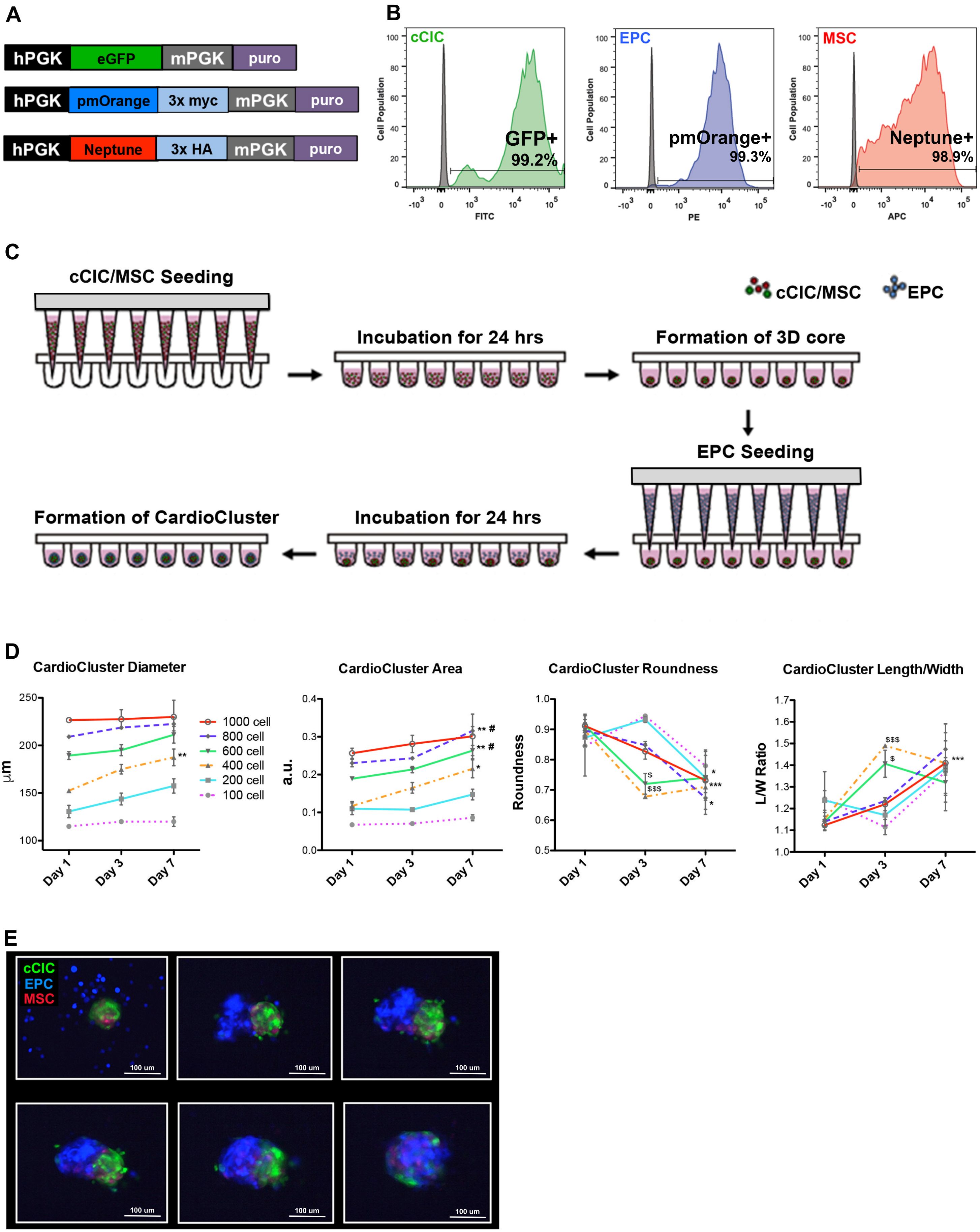
A
hPGK
eGFP
mPGK
puro
hPGK
pmOrange
3x myc
mPGK
puro
hPGK
Neptune
3x HA
mPGK
puro
B
cCIC
GFP+
99.2%
FITC
EPC
pmOrange+
99.3%
PE
MSC
Neptune+
98.9%
APC
Cell Population
C
cCIC/MSC Seeding
cCIC/MSC
EPC
Incubation for 24 hrs
Formation of 3D core
EPC Seeding
Formation of CardioCluster
Incubation for 24 hrs
D
CardioCluster Diameter
µm
1000 cell
800 cell
600 cell
400 cell
200 cell
100 cell
CardioCluster Area
a.u.
CardioCluster Roundness
Roundness
CardioCluster Length/Width
L/W Ratio
Day 1
Day 3
Day 7
E
cCIC
EPC
MSC
100 um

Supplementary Figure 2. CardioCluster formation and characterization

A, Human phosphor-glycerate kinase (hPGK) lentiviral backbones of fluorescent protein tags used to transduce parental cell lines. cCIC express the transgene eGFP, EPC express the transgene mOrange (with a 3x myc tag), and MSC express the transgene Neptune (with a 3x HA tag). **B**, Representative flow cytometry plots showing the percentage of cells expressing their respective fluorescent proteins. **C**, CardioClusters are formed using 96 well, ultra-low attachment round bottom plates in a two-step process. The first step generates the inner core composed of cCIC and MSC, and the second step forms the outer EPC layer. The inner core of cCIC and MSC is seeded for 24 hours. The EPC are added the following day and resulting cell mixture is incubated for an additional 48-72 hours prior to experimentation. **D**, CardioCluster morphometric parameters measuring area (a.u.: arbitrary units.), roundness, and length-to-width (L/W) ratio over a 7 day time course for CardioClusters ranging from 100-1000 cells (n=6 [100 cell], n=9 [200 cell], n=9 [400 cell), n=9 [600 cell], n=8 [800 cell], n=7 [1000 cell] CardioClusters per cell number group). Data represents mean ± SEM. Data are presented as 1-way ANOVA with Tukey's multiple comparisons test, **P=0.005 (diameter, 400 cell); *P=0.013 (area, 400 cell), $^{\#}$P=0.019 (area, 600 cell), **P=0.002 (area, 600 cell), $^{\#}$P=0.011 (area, 800 cell), **P=0.003 (area, 800 cell); ***P<0.001 (roundness, 400 cell), $^{\$}$P=0.017 (roundness, 600 cell), *P=0.046 (roundness, 600 cell), *P=0.021 (roundness, 800 cell); ***P<0.001 (L/W, 400 cell), *P=0.020 (L/W, 600 cell), $^{\$}$ day 1 versus day 3, $^{\#}$ day 3 versus day 7, * day 1 versus day 7. **E**, Still frame images from a video showing CardioCluster formation. Scale Bars, 100 μm.

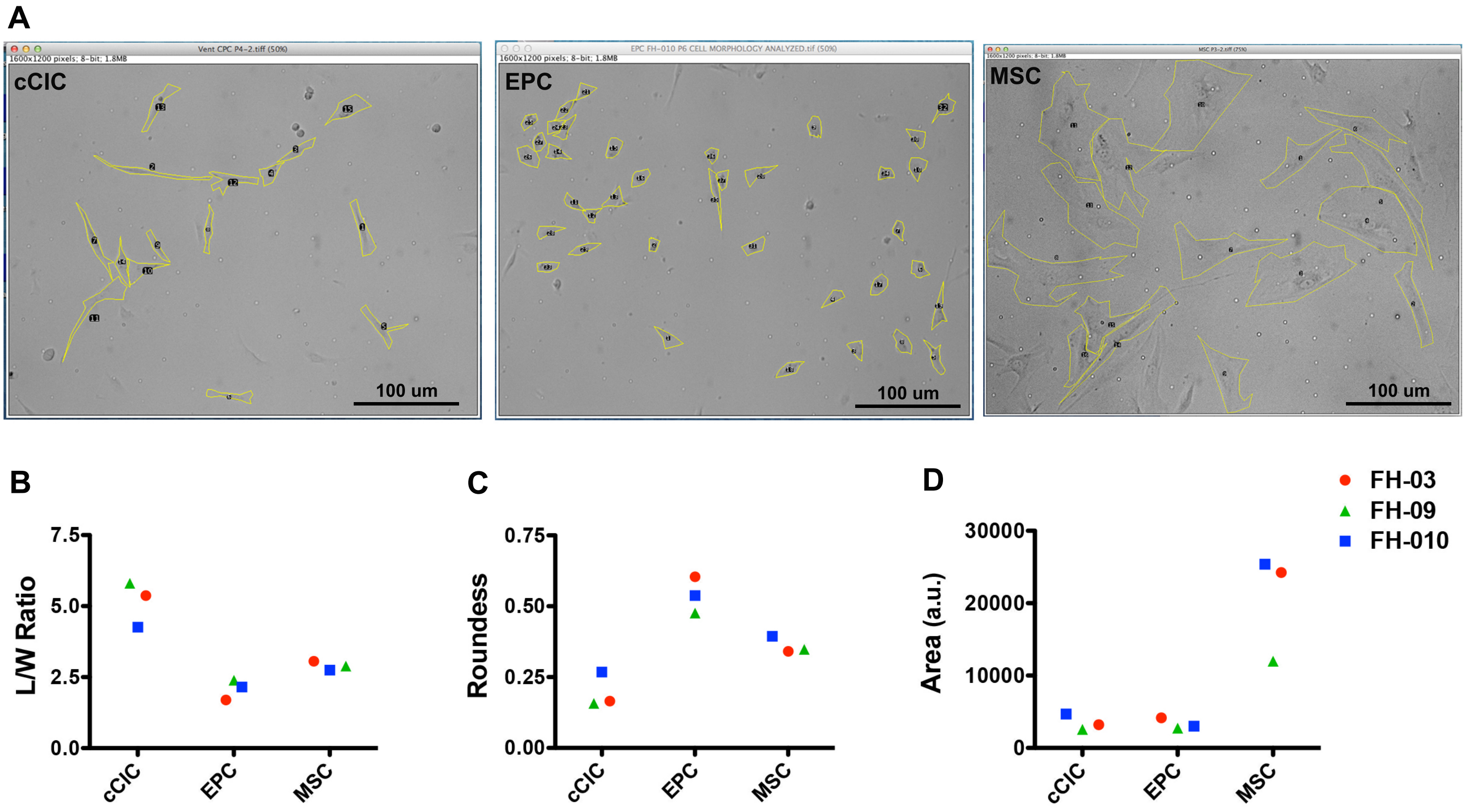

Supplementary Figure 3. Cell morphology measurements
A, Representative phase contrast images of individually traced cells from the three cell populations isolated from human heart samples. Scale Bars, 100 µm. B-D, Scatter plots showing individual heart averages for the morphometric parameters of length-to-width (L/W) ratio **(B)**, roundness **(C)**, and area (arbitrary units [a.u.]) **(D)** (n=3 heart samples, minimum of 30 cells traced per cell type, per patient).

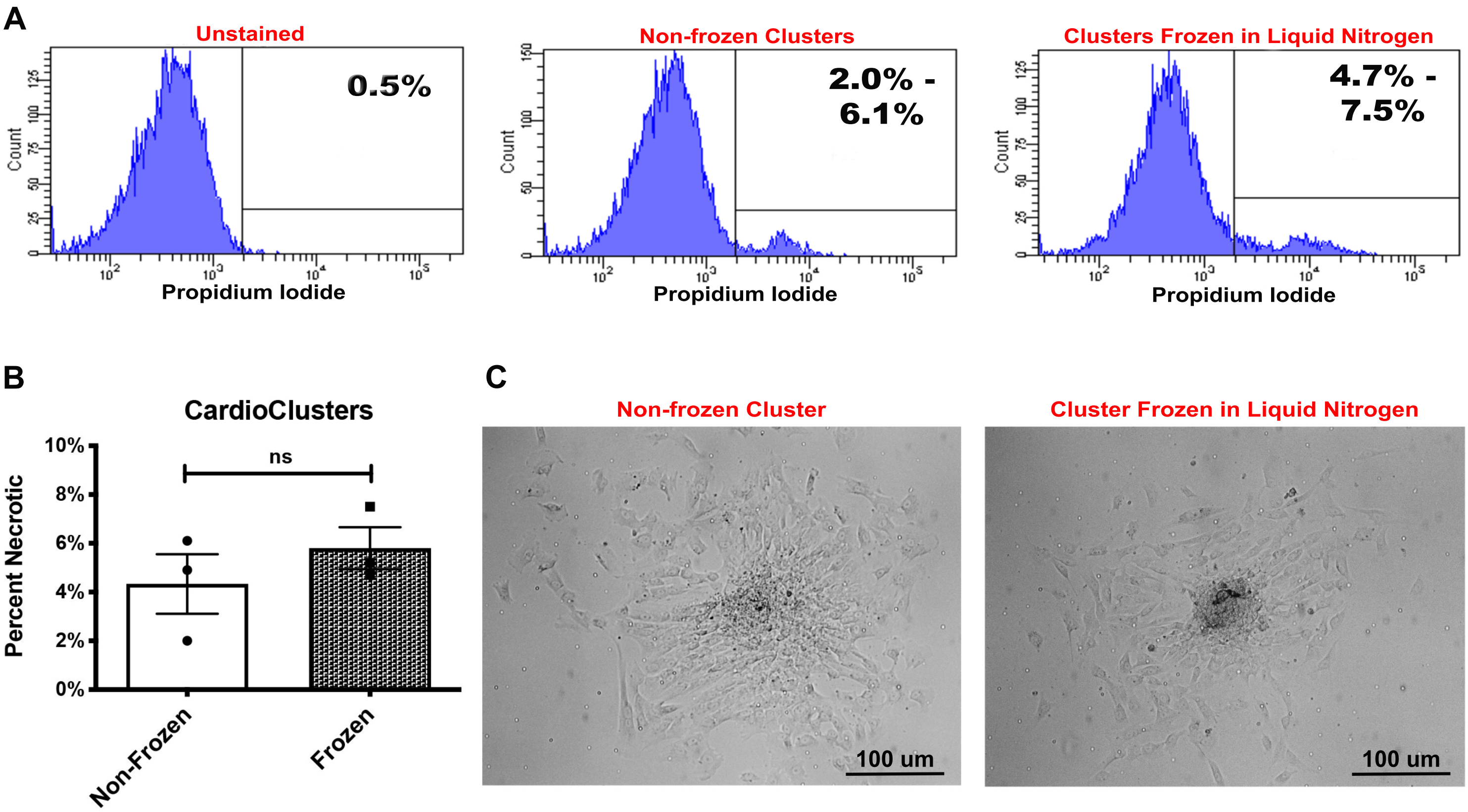

Supplementary Figure 4. CardioClusters frozen in liquid nitrogen maintain structural integrity and viability
A, Representative flow cytometry plots showing propidium iodide (PI) gating strategy used in freezing assay. **B**, Quantification of percent necrotic (PI^+) cells from non-frozen versus liquid nitrogen frozen experimental groups. Data represents mean (n=3 independent experiments, an entire 96-well plate of CardioClusters per experimental group) ± SEM. Data are presented as unpaired t test, versus non-frozen CardioClusters. ns indicates not statically significant. **C**, Brightfield images showing cell outgrowth of non-frozen versus liquid nitrogen frozen CardioClusters. Scale Bars, 100 μm.

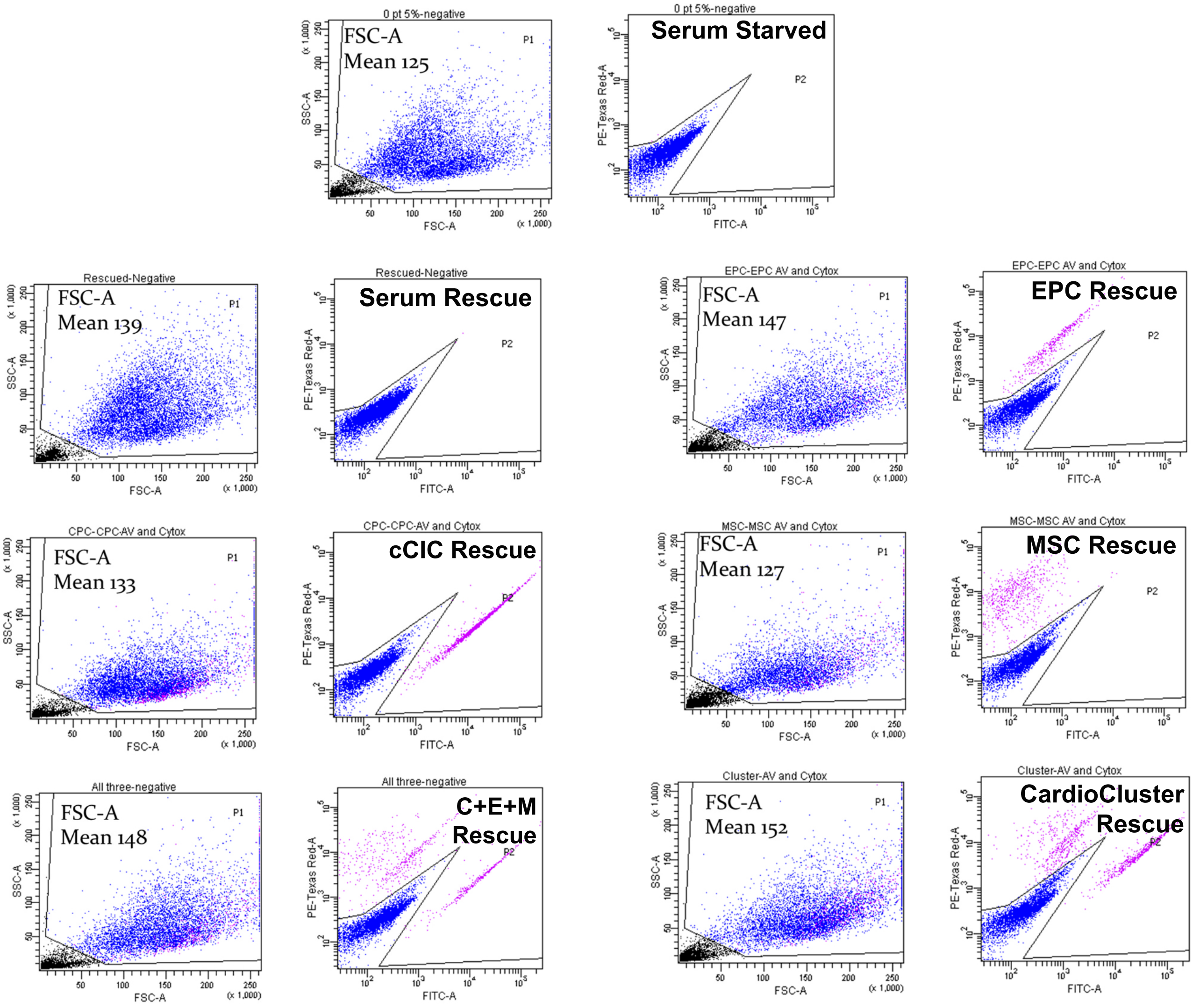

Supplementary Figure 5. CardioClusters restore NRCM morphology following serum starvation
Representative flow cytometry plots showing forward scatter (FSC-A) used to quantitate neonatal rat cardiomyocyte (NRCM) mean area. CIC populations are excluded from analysis by gating out fluorescently tagged cells (represented by pink cells in plots). NRCMs included in analysis are represented in blue.

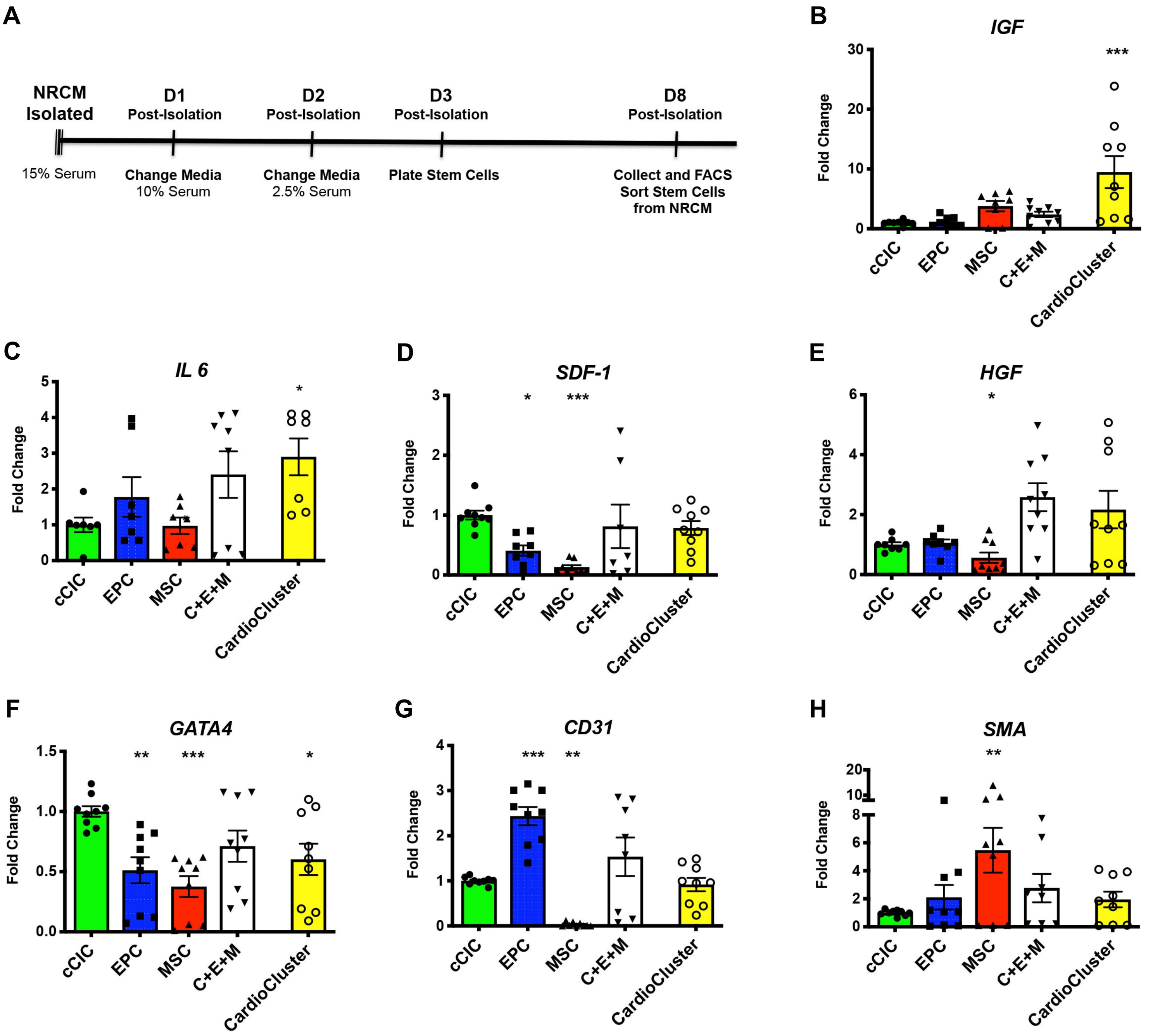

Supplementary Figure 6. CardioClusters show increased paracrine gene expression and remain uncommitted towards a particular lineage after in vitro co-culture with cardiomyocytes

A, Timeline for NRCM co-culture commitment assay. **B-H**, Gene expression in stem cells after a 7-day co-culture with NRCMs. **B**, IGF **C**, IL6 **D**, SDF-1 **E**, HGF **F**, GATA4 **G**, CD31 and **H**, SMA gene expression. Data represent mean (n=4 NRCM preps, each experiment run in triplicate) ± SEM. Data are presented as 1-way ANOVA with Dunnett's comparison test, (**B**) ***p<0.001, (**C**) *p=0.033, (**D**) *p=0.034, ***p<0.001, (**E**) *p= 0.020, (**F**) *p=0.036, **p=0.008, ***p<0.001, (**G**) **p=0.007, ***p<0.001, (**H**) **p=0.007, versus cCIC.

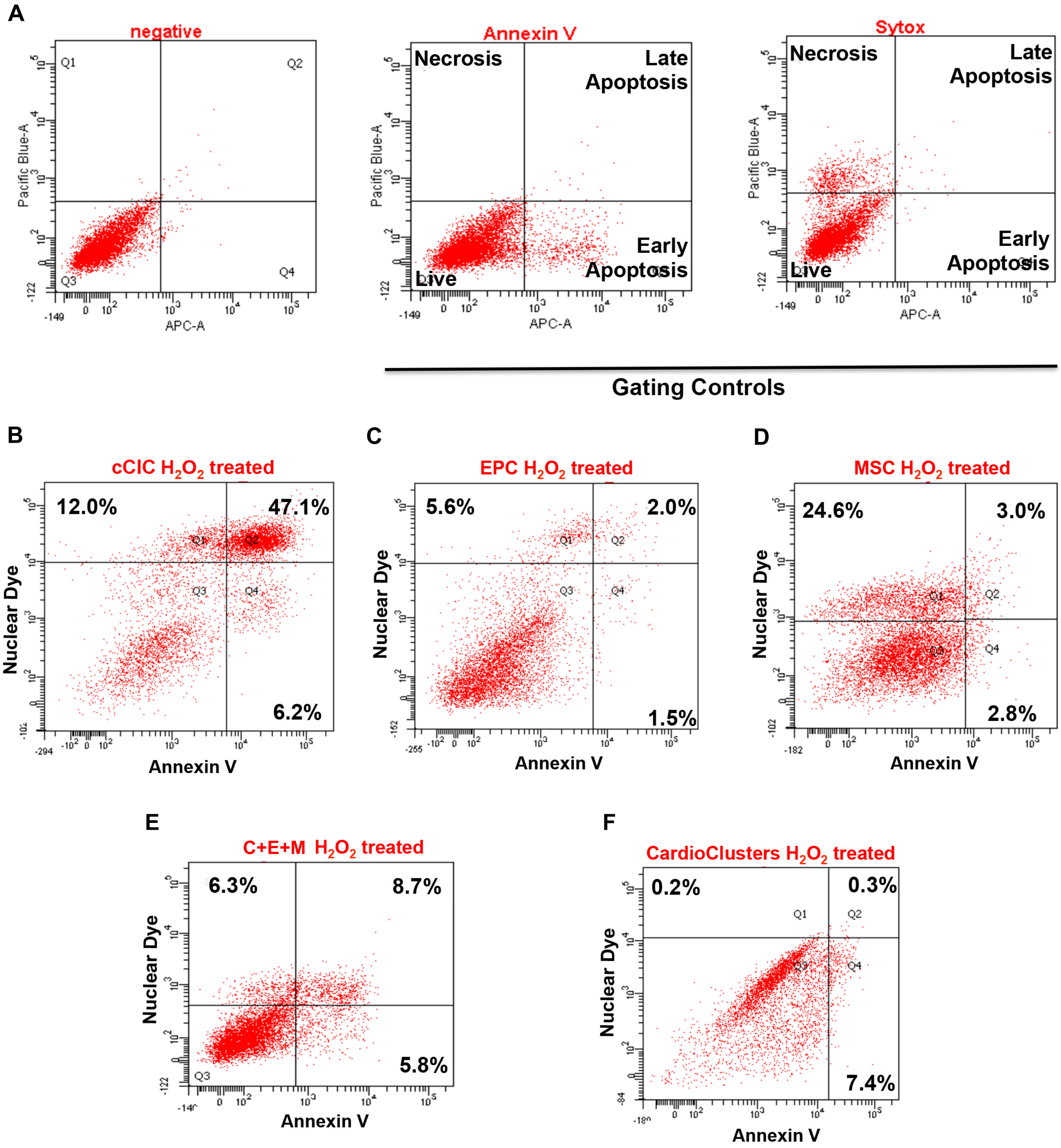

Supplementary Figure 7. Representative staining for markers of apoptosis and necrosis
A, Representative flow cytometry plots showing Annexin V/Sytox Blue gating strategy used in cell death assay. **B-F**, Representative flow cytometry plots showing Annexin V/Sytox Blue labeling following cell death assay on cardiac cell populations under 24 hours of low serum (75% serum reduction) and 4 hours of treatment with 30 μM H_2O_2 in low serum medium for cCIC **(B)**, EPC **(C)**, MSC **(D)**, C+E+M **(E)**, and CardioCluster **(F)**.

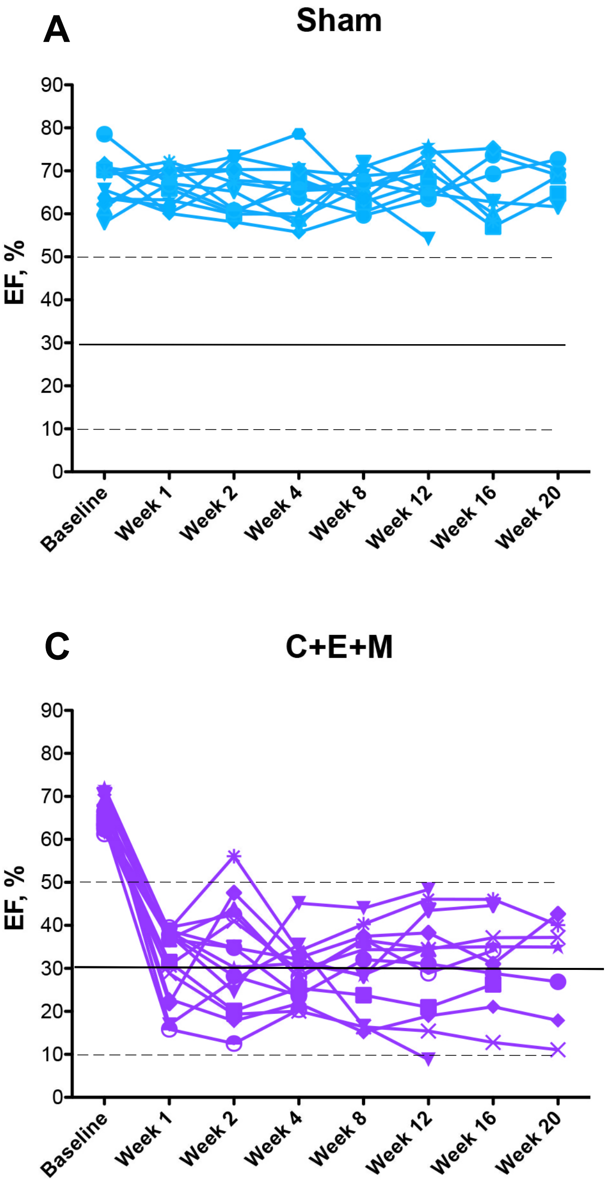

Supplementary Figure 8. Ejection fraction for individual mice grouped by surgery

A-D, Longitudinal assessment of ejection fraction (EF, %) over 20 weeks for individual mice by surgery type: sham **(A)**, vehicle **(B)**, C+E+M **(C)**, and CardioCluster **(D)**.

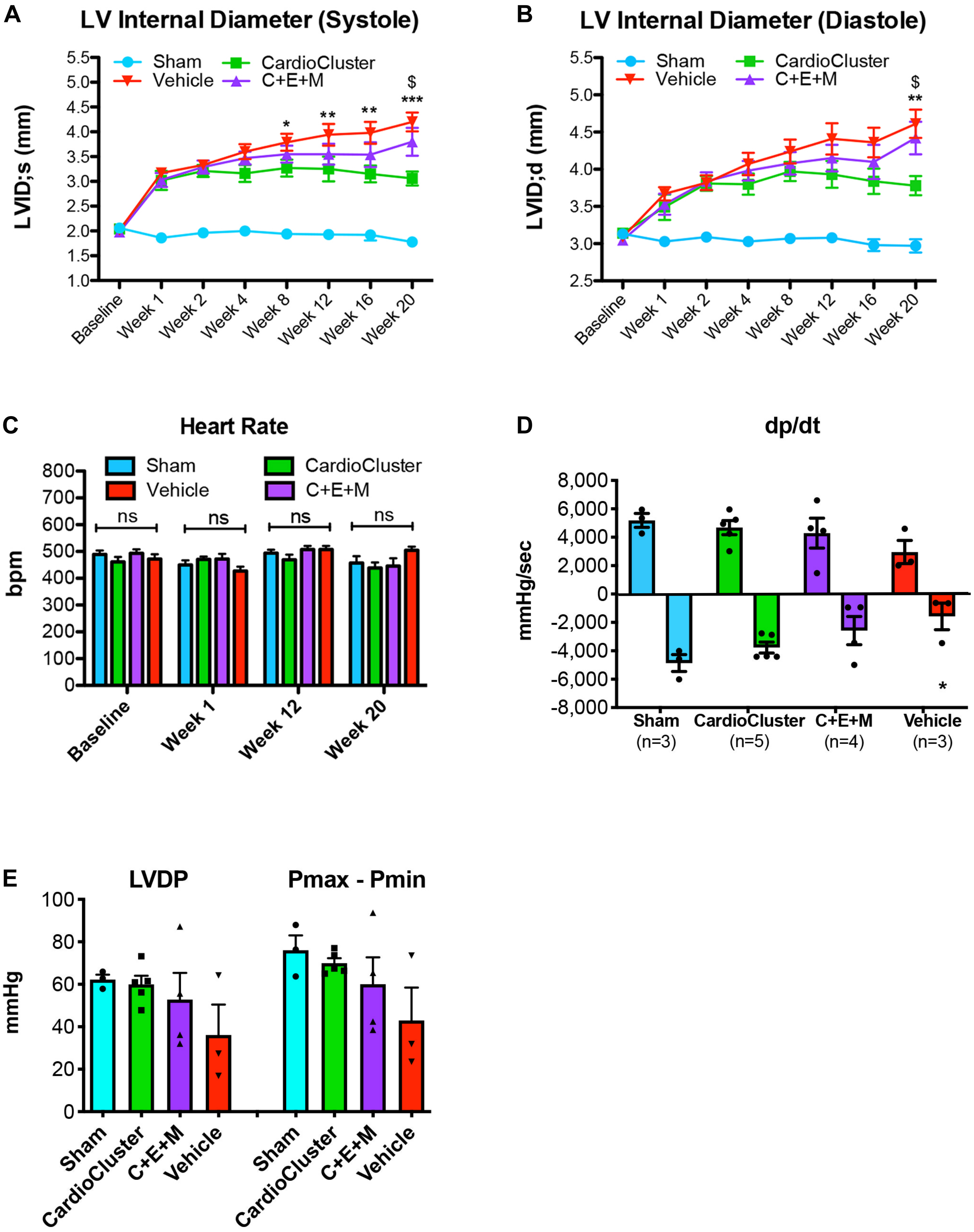
A
LV Internal Diameter (Systole)
Sham
Vehicle
CardioCluster
C+E+M
LVID;s (mm)
$
***
**
*
Baseline
Week 1
Week 2
Week 4
Week 8
Week 12
Week 16
Week 20
B
LV Internal Diameter (Diastole)
LVID;d (mm)
C
Heart Rate
bpm
ns
D
dp/dt
mmHg/sec
Sham (n=3)
CardioCluster (n=5)
C+E+M (n=4)
Vehicle (n=3)
E
LVDP
Pmax - Pmin
mmHg

Supplementary Figure 9. Echocardiographic and hemodynamic data confirms CardioCluster treatment preserves cardiac function

A-B, LV internal diameter (LVID) in systole (LVID;s; **A**) and diastole (LVID;d; **B**). Sample size specified in Supplementary Data 3. Data are presented as 2-way ANOVA with Tukey's multiple comparison test, (**A**) *p=0.045, week 8; **p=0.008, week 12; **p=0.006, week 16; ***p<0.001, week 20, $^{\$}$p=0.033, week 20 and (**B**) **p=0.003, week 20, $^{\$}$p=0.044, week 20. * CardioCluster versus vehicle, $^{\$}$ CardioCluster versus C+E+M. **C,** Heart rates (beats per minute [bpm]) for sham, CardioCluster, C+E+M, and vehicle treatment groups shown at baseline, week 1, week 12 and week 20. **A-C**, Data represents mean ± SEM. **D-E**, Hemodynamic analysis showing developed pressure over time (dP/dt, mmHg/sec; **D**) and left ventricular developed pressure (LVDP, mmHg) and pressure max minus pressure min (P_{max}-P_{min}, mmHg) shown at week 20 (**E**). Data represents mean (n=5 CardioCluster-treated mice; n=4 C+E+M-treated mice; n=3 vehicle-treated mice; n=3 sham) ± SEM. Data are presented as 1-way ANOVA with Dunnett's comparison test, *p=0.04, vehicle versus sham.

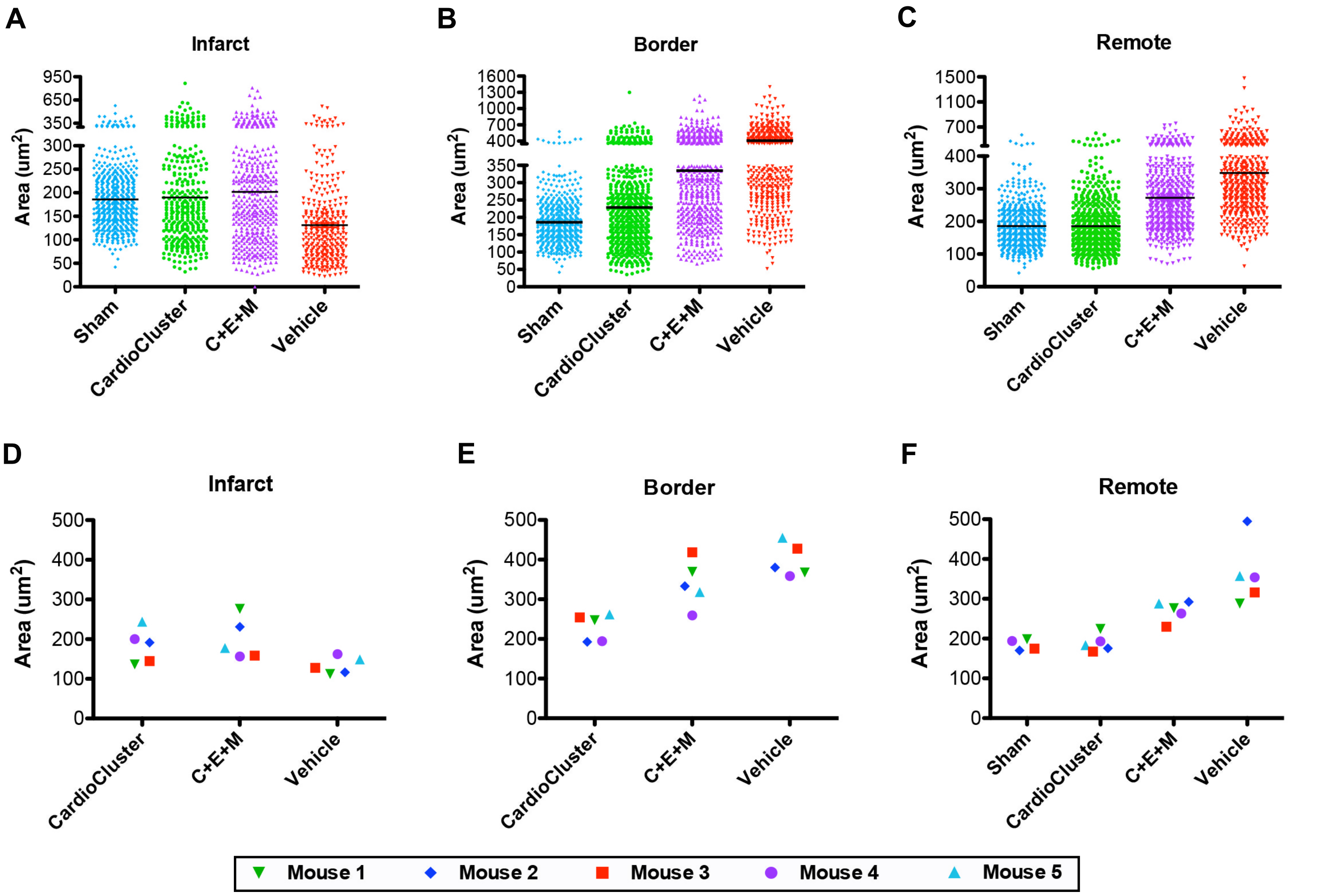

Supplementary Figure 10. CardioCluster treatment antagonizes cardiomyocyte hypertrophy in the border and remote region and preserves cardiomyocyte size in the infarct region

A-C, Scatter plots showing cardiomyocyte cross-sectional area for each individually traced cell in the infarct **(A)**, border **(B)**, and remote **(C)** heart regions. **D-F**, Individual mean for each mouse used to quantify cardiomyocyte cross-sectional area in the infarct **(D)**, border **(E)**, and remote **(F)** heart regions shown by scatter plots (n=5 CardioCluster-treated mice; n=5 C+E+M-treated mice; n=5 vehicle-treated mice; n=4 sham mice).

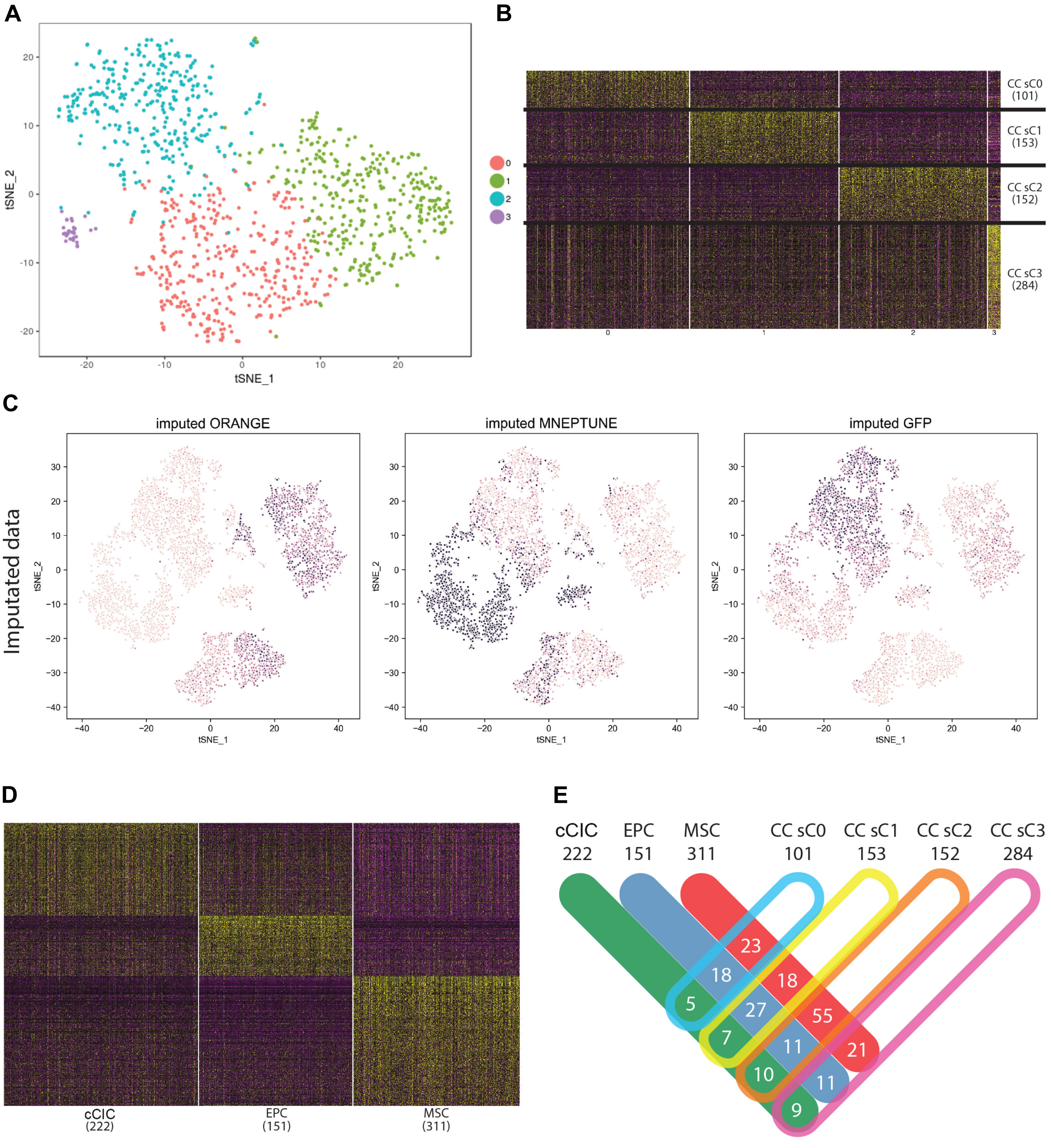

Supplementary Figure 11. Internal heterogeneity within a CardioCluster

A, t-SNE of CardioClusters internal transcriptional groups. B, A heatmap of the DEGs found within the four sub-clusters of a CardioCluster. **C**, Transcriptional imputation of reporter fluorophores reveals vestigial similarities between the individual cells within a CardioCluster and parental populations. **D**, Transcriptional signatures of parental populations is revealed by DEGs within a heatmap. **E**, Transcriptional intersectionality reveals vestigial similarities of CardioCluster sub-clusters (CC sC) to primarily EPC/MSC parental populations shown by a Venn Diagram displaying the overlap of transcriptional signatures.

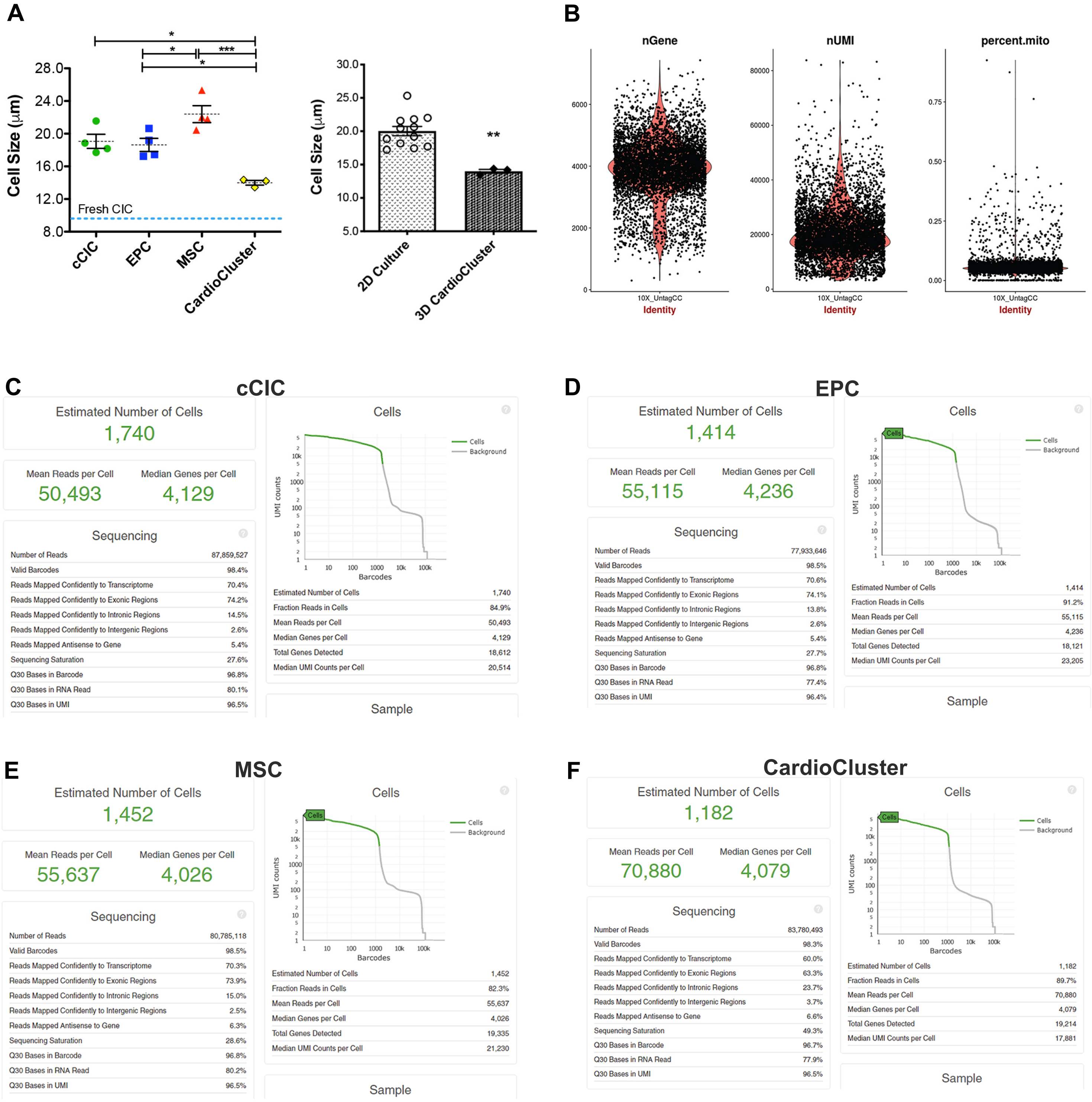

Supplementary Figure 12. Quality control for single-cell RNA sequencing

A, Cell size quantification for average diameter of 2D cultured cCICs, EPCs, and MSCs versus cells cultured within a 3D CardioCluster. Data represents mean (n=4 cCIC, n=4 EPC, n=4 MSC, n=3 CardioCluster) ± SEM. Left graph, data are presented as 1-way ANOVA with Tukey's multiple comparisons test, **P=0.009, cCIC versus CardioCluster, *p=0.035, EPC versus MSC, *p=0.017, EPC versus CardioCluster, ***p<0.001, MSC versus CardioCluster. Right graph data are presented as unpaired t test, **P=0.001. **B**, Violin plots for the number of genes, unique molecular identifiers (UMIs) and percent mitochondrial genes used for single-cell RNA sequencing quality control. **C**-**F**, Cell Ranger 2.0 quality control summary for cCIC **(C)**, EPC **(D)**, MSC **(E)** and CardioCluster **(F)**.
