# Supplementary material for: Enhancing myocardial repair with CardioClusters
Source: Nat Commun. 2020 Aug 7;11:3955. doi: 10.1038/s41467-020-17742-z (PMC7414230; doi:10.1038/s41467-020-17742-z)
Supplement: Supplementary file 3 — Description of Additional Supplementary Files [file 41467_2020_17742_MOESM3_ESM.docx]

| File Name: **Supplementary Data 1** |
| --- |
| Description: Dataset of DEGs enriched in cCICs, EPCs, MSCs and CardioClusters. |
| File Name: **Supplementary Data 2** |
| Description: Dataset of DEGs enriched in freshly isolated cardiac interstitial cells versus CardioClusters and parental 2D cultures. |
| File Name: **Supplementary Data 3** |
| Description: Heart rate and echocardiographic data. Echocardiographic data represented as mean ± SEM. Heart rate, anterior wall thickness, posterior wall thickness, left ventricular volume, ejection fraction and fractional shortening were measured at specified times after MI. (N) indicates the number of mice used in each group at the given time point. |
| File Name: **Supplementary Video 1** |
| Description: CardioClusters spontaneous self-assembly revealed using time-lapse video microscopy. |
| File Name: **Supplementary Video 2** |
| Description: CardioClusters spontaneous self-assembly with changed seeding sequence so that cCIC+EPCs are added prior to MSC seeding. |
