# Supplementary material for: Enhancing myocardial repair with CardioClusters
Source: Nat Commun. 2020 Aug 7;11:3955. doi: 10.1038/s41467-020-17742-z (PMC7414230; doi:10.1038/s41467-020-17742-z)
Supplement: Supplementary file 9 — Reporting Summary [file 41467_2020_17742_MOESM9_ESM.pdf]

nature research

Corresponding author(s): Mark A Sussman

Last updated by author(s): 6/16/2020

Reporting Summary

Nature Research wishes to improve the reproducibility of the work that we publish. This form provides structure for consistency and transparency in reporting. For further information on Nature Research policies, see Authors & Referees and the Editorial Policy Checklist .

Statistics

For all statistical analyses, confirm that the following items are present in the figure legend, table legend, main text, or Methods section.

n/a	Confirmed	
☐	☒	The exact sample size (*n*) for each experimental group/condition, given as a discrete number and unit of measurement
☐	☒	A statement on whether measurements were taken from distinct samples or whether the same sample was measured repeatedly
☐	☒	The statistical test(s) used AND whether they are one- or two-sided<br>*Only common tests should be described solely by name; describe more complex techniques in the Methods section.*
☒	☐	A description of all covariates tested
☐	☒	A description of any assumptions or corrections, such as tests of normality and adjustment for multiple comparisons
☐	☒	A full description of the statistical parameters including central tendency (e.g. means) or other basic estimates (e.g. regression coefficient) AND variation (e.g. standard deviation) or associated estimates of uncertainty (e.g. confidence intervals)
☐	☒	For null hypothesis testing, the test statistic (e.g. *F*, *t*, *r*) with confidence intervals, effect sizes, degrees of freedom and *P* value noted<br>*Give P values as exact values whenever suitable.*
☒	☐	For Bayesian analysis, information on the choice of priors and Markov chain Monte Carlo settings
☒	☐	For hierarchical and complex designs, identification of the appropriate level for tests and full reporting of outcomes
☒	☐	Estimates of effect sizes (e.g. Cohen's *d*, Pearson's *r*), indicating how they were calculated

Our web collection on statistics for biologists contains articles on many of the points above.

Software and code

Policy information about availability of computer code

Data collection	Flow cytometry was collected with a BD FACS Canto and BD FACSAria instrument (BD Biosciences). q-RT PCR was run using a CFX Real-Time PCR Detection System (Bio-Rad Laboratories). Transthoracic echocardiography was performed using a Vevo 2100 (VisualSonics). Invasive hemodynamic data acquisition was performed with an ADVantage PV System (ADV500, Transonic Systems Inc.) using a 1.2F PV catheter (Transonic Systems Inc.). A Leica DMIL6000 microscope and Leica TCS SP8 Confocal Microscope were used.
Data analysis	Flow cytometry data was analyzed by Flow Jo software (BD Biosciences). Strain analysis was conducted using a speckle-tracking algorithm provided by VisualSonics (VevoStrain). Hemodynamic data analysis was performed by LabScribe v3 software (iWorx). Image J software was used for section quantification and cell morphology measurements. Leica LAS X analysis software and the drawing tool in the SP8 TCS Leica Software were both used for analysis. Statistical analysis was performed using GraphPad Prism version 5.0 and 8.0 software. Gene expression pathway analysis was performed using R package clusterProfiler.

For manuscripts utilizing custom algorithms or software that are central to the research but not yet described in published literature, software must be made available to editors/reviewers. We strongly encourage code deposition in a community repository (e.g. GitHub). See the Nature Research guidelines for submitting code & software for further information.

Data

Policy information about availability of data

All manuscripts must include a data availability statement. This statement should provide the following information, where applicable:

- Accession codes, unique identifiers, or web links for publicly available datasets
- A list of figures that have associated raw data
- A description of any restrictions on data availability

scRNA-Seq data generated for CardioClusters, CICs, EPCs, and MSCs was uploaded to the Gene Expression Omnibus (GEO submission GSE133832, released July 4, 2019). Datasets for freshly isolated mouse CICs have been previously published by our group and are available at the GEO database (accession number GSE114280).

Field-specific reporting

Please select the one below that is the best fit for your research. If you are not sure, read the appropriate sections before making your selection.

- [x] Life sciences
- [ ] Behavioural & social sciences
- [ ] Ecological, evolutionary & environmental sciences

For a reference copy of the document with all sections, see nature.com/documents/nr-reporting-summary-flat.pdf

Life sciences study design

All studies must disclose on these points even when the disclosure is negative.

Sample size

The statistical method used to predetermine the appropriate *in vivo* animal sample size was by an online sample size calculator (http://www.lasec.cuhk.edu.hk/sample-size-calculation.html). For *in vitro* experiments sample size was performed with a minimum of three independent experiments run in duplicate or triplicate. This number of independent experiments is required to perform statistical analysis.

Data exclusions

For mice to be included in this study, it was pre-established that ejection fraction (EF) had to drop below 50% at the 1-week time point following infarction injury, mice above 50% were excluded. The reason being that we wanted a uniform cohort of mice which excluded mice whose infarcts were too small.

Replication

Each experimental protocol was successfully replicated a minimum of three times.

Randomization

All samples were randomly selected and assigned to each group for analysis.

Blinding

For in vivo animal experiments, investigators were blinded to surgical procedures, injections, and analysis. Blinding was not feasible for most *in vitro* experiments because implementation required treatment groups to be unmasked to the investigator as part of the experimental design for proper execution. However, objectivity was maintained at all times and at the highest level in recording the outcome assessments. Outcomes were reliable, there were multiple assessments of outcomes, and a high levels of concurrence between multiple assessors, as well as use of instrumentation in some cases that provided unbiased quantitation. Collectively, these measures mitigate potential for bias influencing data analysis and interpretation.

Reporting for specific materials, systems and methods

We require information from authors about some types of materials, experimental systems and methods used in many studies. Here, indicate whether each material, system or method listed is relevant to your study. If you are not sure if a list item applies to your research, read the appropriate section before selecting a response.

Materials & experimental systems

n/a	Involved in the study	
[ ]	[x]	Antibodies
[ ]	[x]	Eukaryotic cell lines
[x]	[ ]	Palaeontology
[ ]	[x]	Animals and other organisms
[x]	[ ]	Human research participants
[x]	[ ]	Clinical data

Methods

n/a	Involved in the study	
[x]	[ ]	ChIP-seq
[ ]	[x]	Flow cytometry
[x]	[ ]	MRI-based neuroimaging

Antibodies

Antibodies used

C-Kit (CD117) R&D systems AF1356
Thy-1 (CD90), Clone 5E10 Biolegend 328109
Endoglin (CD105) Biolegend 323203
Prominin-1 (CD133) Thermo Fisher Scientific PA5-38014
PTPRC (CD45) Biolegend 368507
cTNT, ALEXA FLUOR® 488 Conjugated Biocompare bs-10648R-A488
Tropomyosin Sigma-Aldrich T 9283
eGFP Molecular Probes A-11122
mCherry Thermo Fisher Scientific M11240
Isolectin GS-IB4, ALEXA FLUOR® 568 Conjugated Thermo Fisher Scientific I21412
WGA Thermo Fisher Scientific W32465
Myc tag Thermo Fisher Scientific PA3-981
HA-prope Santa Cruz Biotechnology SC-7392
DAPI (4,6-diamidino-2-phenylindole) Sigma-Aldrich D9542
Phalloidin Thermo Fisher Scientific A12379

Validation

Each of these antibodies was validated by vendor:
CD117/c-kit was detected in immersion fixed frozen sections of mouse embryo at 15 µg/mL overnight at 4 °C. Also validated for flow cytometry on lineage depleted mouse bone marrow cells.
Thy-1 (CD90) in human erythroleukemic cell line HEL stained for flow cytometry.
Endoglin (CD105) in human monocytic cell line THP-1 stained for flow cytometry.
CD133/Prominin-1 immunofluorescent analysis of Jurkat cells using the polyclonal antibody at a dilution of 20 µg/mL.
PTPRC (CD45) flow cytometry analysis using Jurkat cells.
Tropomyosin minimum titer of 1:50 was determined by staining of formalin-fixed, paraffin-embedded human tissue sections.
eGFP tested by western blot analysis of HeLa whole cell lysate versus GFP-transfected HeLa cell lysates.
mCherry tested by flow cytometry of cells stained with mCherry rat monoclonal antibody.

Each of these antibodies was validated in house:
cTNT, ALEXA FLUOR® 488 Conjugated tested on mouse tissue sections.
Isolectin GS-IB4, ALEXA FLUOR® 568 Conjugated tested on mouse tissue sections.
WGA, DAPI, and Phalloidin were validated on mouse tissue sections.
Myc and Ha tags were validated by western blots of cardiac cell lines transduced with lentivirus to express each of the tags.

Eukaryotic cell lines

Policy information about cell lines

Cell line source(s)	Cells were derived from primary tissue explands from hearts of post-mortem infants provided by a commercial source (Novogenix Laboratories).
Authentication	Surface marker characteristics, growth characteristics, differentiation assays, and cell morphological analysis were performed to validate cell lines.
Mycoplasma contamination	Not tested for mycoplasma.
Commonly misidentified lines (See ICLAC register)	No commonly misidentified lines were use.

Animals and other organisms

Policy information about studies involving animals; ARRIVE guidelines recommended for reporting animal research

Laboratory animals	8-week old NOD.CB17-Prkdcscid/J female mice (The Jackson Laboratory, catalog #001303)
Wild animals	No wild animals were used.
Field-collected samples	No field-collected samples were used.
Ethics oversight	All procedures and experiments involving mice were conducted by observing ethical guidelines for animal studies as approved by the SDSU Institutional Animal Care and Use Committee.

Note that full information on the approval of the study protocol must also be provided in the manuscript.

Flow Cytometry

Plots

Confirm that:

- [x] The axis labels state the marker and fluorochrome used (e.g. CD4-FITC).
- [x] The axis scales are clearly visible. Include numbers along axes only for bottom left plot of group (a 'group' is an analysis of identical markers).
- [x] All plots are contour plots with outliers or pseudocolor plots.
- [x] A numerical value for number of cells or percentage (with statistics) is provided.

Methodology

Sample preparation	For cell analysis cultured cells with collected and antibody labeled either live or following fixation in 4% paraformaldehyde for five minutes at room temperature.
Instrument	BD FACSCanto and BD FACSAria
Software	Flow-Jo
Cell population abundance	10,000 events per sample were recorded
Gating strategy	Gating was set based on secondary antibody, or isotype controls when commercially available. Background was gated to 2% or less based on secondary control.

- [x] Tick this box to confirm that a figure exemplifying the gating strategy is provided in the Supplementary Information.
